# Structural and Functional Substitution of Deleted Primary Sensory Neurons by New Growth from Intrinsic Spinal Cord Nerve Cells: An Alternative Concept in Reconstruction of Spinal Cord Circuits

**DOI:** 10.3389/fneur.2017.00358

**Published:** 2017-07-24

**Authors:** Nicholas D. James, Maria Angéria, Elizabeth J. Bradbury, Peter Damberg, Stephen B. McMahon, Mårten Risling, Thomas Carlstedt

**Affiliations:** ^1^The Wolfson Centre for Age-Related Diseases, King’s College London, London, United Kingdom; ^2^Department of Neuroscience, Karolinska Institutet, Solna, Sweden; ^3^Department of Clinical Neuroscience, Karolinska Institutet, Solna, Sweden

**Keywords:** avulsion injury, sensory neurons, plasticity, proprioception, electrophysiology

## Abstract

In a recent clinical report, return of the tendon stretch reflex was demonstrated after spinal cord surgery in a case of total traumatic brachial plexus avulsion injury. Peripheral nerve grafts had been implanted into the spinal cord to reconnect to the peripheral nerves for motor and sensory function. The dorsal root ganglia (DRG) containing the primary sensory nerve cells had been surgically removed in order for secondary or spinal cord sensory neurons to extend into the periphery and replace the deleted DRG neurons. The present experimental study uses a rat injury model first to corroborate the clinical finding of a re-established spinal reflex arch, and second, to elucidate some of the potential mechanisms underlying these findings by means of morphological, immunohistochemical, and electrophysiological assessments. Our findings indicate that, after spinal cord surgery, the central nervous system sensory system could replace the traumatically detached original peripheral sensory connections through new neurite growth from dendrites.

## Introduction

The first human case in which spinal cord surgery resulted in the return of sensorimotor function and the restoration of a reflex arch after spinal root avulsion, in conjunction with a brachial plexus lesion, was recently presented (1). The root avulsion injury is a spinal cord (or central nervous system, CNS) lesion which was not previously considered amenable to treatment, as regeneration of new nerve fibers has to occur in CNS tissue of the spinal cord.

Reimplanting avulsed motor roots into the spinal cord can restore muscle function. Recovery is explained by the original findings that, as a first critical and crucial part of recuperation, spinal cord motorneurons can regrow within the CNS as proper CNS regeneration (2). This unique finding resulted in a surgical technique that is now an established clinical treatment and can restore useful motor (but not sensory) function in patients, even those with the most severe and complicated brachial plexus avulsion injuries (3–5).

In contrast to the ability of motor neurons to regrow within, and extend beyond, the spinal cord after root avulsion or intraspinal injury, it is well documented that sensory nerve fibers cannot regenerate back into the spinal cord after injury (6, 7). Obviously, sensory function cannot be recovered by reimplanting avulsed sensory roots in these injuries, which for the affected patients means agonizing chronic pain, loss of proprioceptive and exteroceptic sensation, as well as reduced muscle coordination and function (5).

Hypothetically, sensory nerve cells in the spinal cord could elongate new processes into a PNS graft implanted into the dorsal spinal cord to reconnect with the periphery. Indeed, medullary implantation of a nerve graft into the dorsal horn of the spinal cord has been documented to incite extension of processes from spinal cord dorsal horn neurons into the sensory part of a spinal nerve after the dorsal root ganglion has been removed, in effect bypassing the primary sensory neurons (8, 9). This original experimental observation led to application in a clinical case, the outcome of which was the return of a spinal cord reflex (1).

The objective of this study is to further elucidate the mechanisms underlying the clinical observation of surgically provoked spinal cord recovery and plasticity of sensory systems. The nature of the *de novo* neurites from the dorsal horn, as well as the ability to electrically evoke local spinal segmental reflexes, is examined. In effect, this is an “inverted” translational study.

## Materials and Methods

### Animals and Injury Model

**Figure 1:**
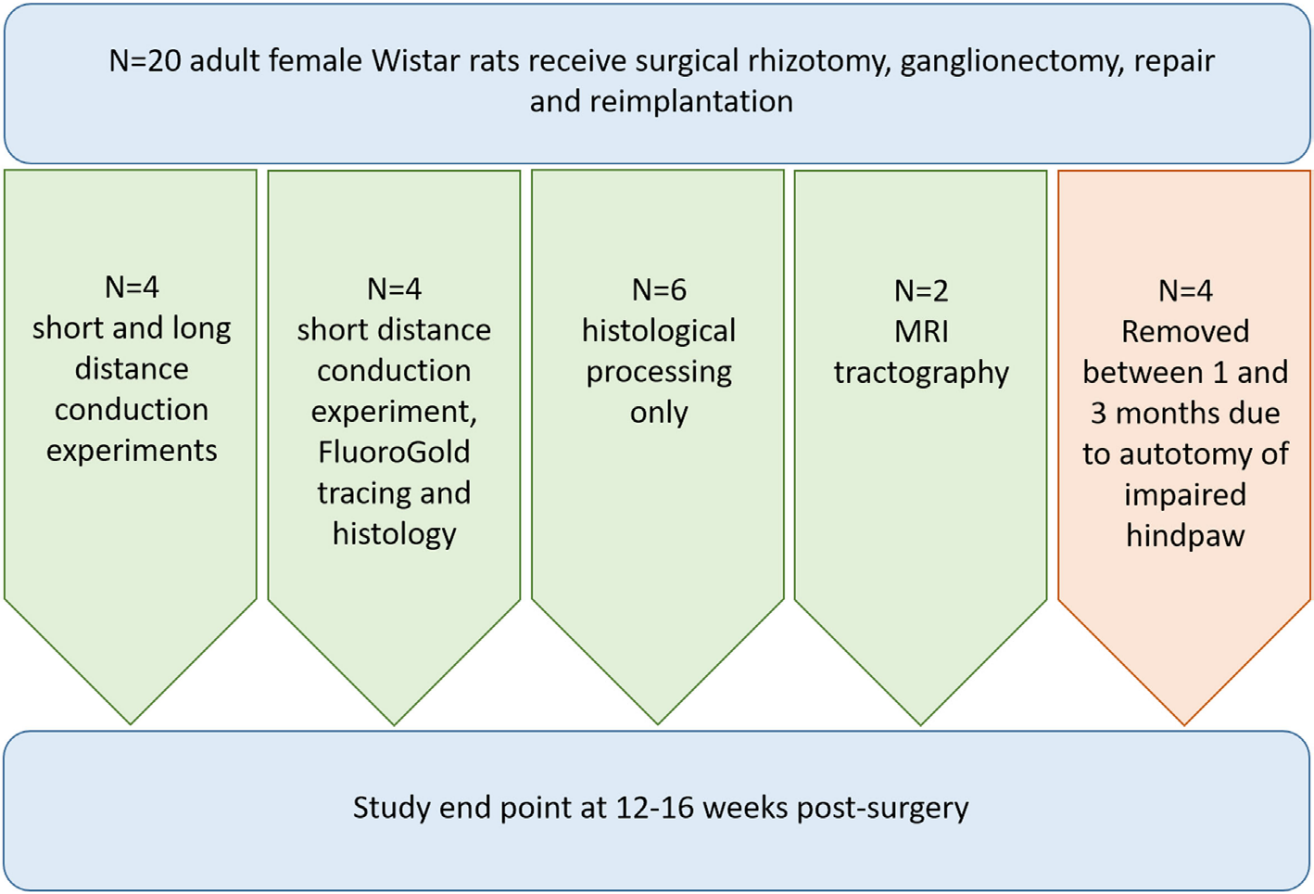
Flowchart illustrating the breakdown of animals used for each outcome measure in the study.

**Figure 2:**
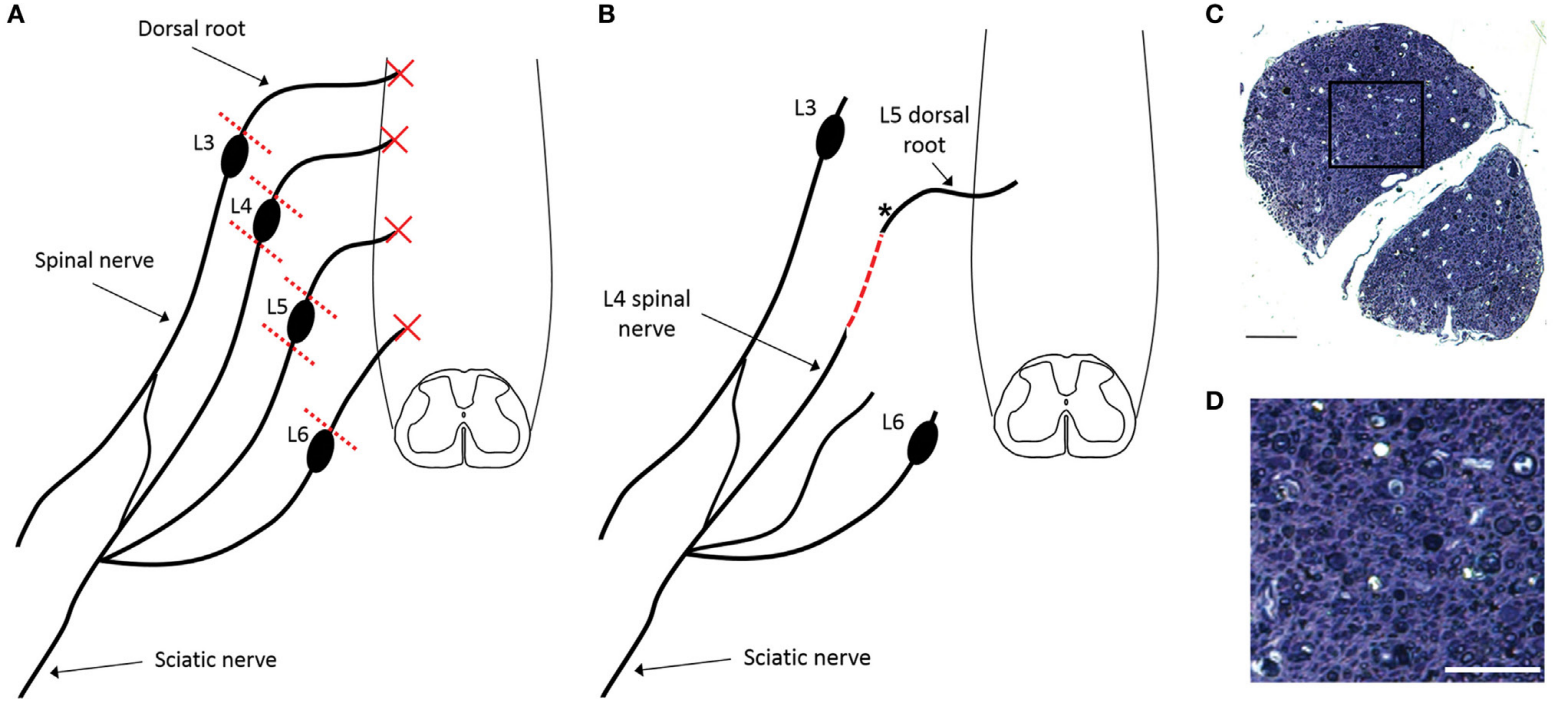
Schematic representation of surgical injury model indicating the rhizotomy of L3–L6 dorsal roots and ganglionectomy of L4 and L5 DRGs **(A)** as well as the subsequent reimplantation of the conjoined L4 spinal nerve and L5 dorsal root **(B)**. Dashed red line indicates the site of surgical repair, where L5 dorsal root transitions into L4 spinal nerve. **(C)** Semi-thin transverse section of plastic-embedded reimplanted dorsal root indicating the presence of numerous small diameter myelinated axons, despite the surgical removal of the dorsal root ganglion [section taken from site marked with asterisk in panel **(B)**]. **(D)** Magnification of boxed area shown in panel **(C)**. Scale bar in panel **(C)** = 50 µm and in panel **(D)** = 25 µm.

All experimental procedures were performed according to United Kingdom Scientific Procedures Act (1986). Figure 1 illustrates the number of animals used for individual outcomes. 20 adult female Wistar rats (220–250 g; Harlan Laboratories) were surgically anesthetized using a mixture of ketamine (60 mg/kg) and medetomidine (0.25 mg/kg; administered i.p.). A skin incision was made medial to the iliac crest and blunt dissection through the longissimus muscle to the left lateral L5 vertebral process was performed. This was removed and a hemi-laminectomy was performed in order to expose the terminal parts of L3–L6 dorsal roots and their associated dorsal root ganglia. The dorsal roots of L3–L6 were cut near the dorsal root ganglia (DRG) and the L4 and L5 DRG were removed entirely (Figure 2A). A second, more rostral, hemi-laminectomy was performed and the L3–L6 dorsal roots were cut flush with the spinal cord surface. The L5 dorsal root was then reimplanted through a small opening in the pia mater just caudal to its original site of attachment with the spinal cord. The distal end of the L5 dorsal root was long enough to then be coapted to the sensory part of the L4 spinal nerve (Figure 2B). The end of the L5 dorsal root and the sensory part of the L4 spinal nerve were held together with Tisseal glue. The wound was then closed in layers. All animals were administered appropriate analgesia at the time of surgical induction, 24, and 48 h post-surgery (carprofen, 5 mg/kg; s.c. delivery).

### Tissue Processing

At 12–16 weeks post-injury terminally anesthetized animals underwent transcardial perfusion with heparinized 0.9% NaCl solution followed by 4% paraformaldehyde in 0.1 M phosphate buffer (PB). The L5 spinal cord segment and the replanted dorsal root were rapidly removed and post fixed with 4% paraformaldehyde in 0.1 M PB for 2 h at room temperature. For spinal cord sections, the spinal cord tissue was embedded in paraffin wax and cut in 25 µm transverse sections using a microtome. For semi-thin reimplanted dorsal root sections, tissue was osmicated and then dehydrated in a graded series of ethanol and then embedded in Durcopan (Fluka, Sigma-Aldrich GmbH). After polymerization, the specimens were cut in 0.5 µm semi-thin sections on a LKB Ultrotome V and stained with toluidine blue. Images were obtained with a NIKON E600 microscope with a 60 × oil immersion plan-apo lens and a Nikon Digital Sight DS-U1 camera or a NIKON EZC1 confocal imaging system.

### Electrophysiology

Electrophysiological assessment of function in reimplanted, ganglionectomised dorsal root/spinal nerves was carried out 12–16 weeks post-surgery on rats (*n* = 8) deeply anesthetized using urethane (1.25 g/kg i.p.). Depth of anesthesia was regularly assessed by monitoring withdrawal reflexes and respiratory rate. Core temperature was maintained at 37°C using a self-regulating heating blanket. Soft tissue was dissected to re-expose the ganglionectomised dorsal root/spinal nerve from its spinal replantation site to the iliac crest. The ganglionectomised dorsal root was hooked onto bipolar silver wire stimulating electrodes just distal to the reimplantation site (~5 mm). The dorsal root was raised well above surrounding tissues on these silver wire hook electrodes and a thin sheet of plastic was placed over the surrounding tissue beneath the electrodes to ensure the stimulus could not spread to any other tissues. Similar bipolar silver wire recording electrodes were placed approximately 15 mm distal to the stimulation site (Figure 4A, “R1”). In *n* = 4 of these rats, the sciatic nerve in the left hindlimb was also exposed at mid-thigh level and placed on bipolar silver wire recording electrodes (Figure 4B, “R2”). When recording activity in the sciatic nerve, the stimulating electrode pair was moved 10 mm distal to the original stimulation location described above (i.e., stimulation now 15 mm, rather than 5 mm, distal to the reimplantation site) in order to ensure there was no spread of stimulation to the spinal cord. All exposed tissues were immersed in mineral oil to prevent current spread and the drying out of nervous tissue. Stimulation intensity was supra-maximal (3 mA, 1 ms duration square wave pulses delivered at a frequency of 0.2 Hz) in order to ensure that even small diameter, non-myelinated fibers would be activated. Recordings were captured using LabChart software (AD Instruments).

### FluoroGold (FG) Injections

One week prior to terminal electrophysiological experiments, *n* = 4 rats received injections of the retrograde tracer FG (Santa Cruz Biotechnology) into the left sciatic nerve. Under gaseous anesthesia (2% isoflurane), the left sciatic nerve was exposed, a small hole was cut in the epineurium using microdissection tools and a 5 µl Hamilton syringe was then inserted through this hole into the nerve tissue and used to slowly deliver 4 µl of 2% FG (in PBS) over the course of 90 s. The Hamilton syringe was left in position for 2 min following injection to minimize any backflow of tracer. Muscle and skin were then sutured separately and animals were administered analgesics (carprofen; 5 mg/kg) and allowed to recover for 2 h in a heated incubator before being returned to their home cage.

### Immunohistochemistry

Sections were incubated in primary antibody in 0.01 M PBS with 5% bovine serum albumin and 0.3% Triton at 4°C overnight. Primary antibody was removed by washing three times for 5 min each in 0.01 M PBS. Sections were then incubated with appropriate secondary antibodies for 2 h at room temperature in 0.01 M PBS with 0.3% triton and 5% normal donkey serum, and then washed in 0.01 M PBS three times for 5 min. Slides were then coverslipped using Mowiol mounting medium. Primary antibodies used were rabbit anti-GFAP (1:2,000, Dako), mouse anti-NeuN (1:500, Millipore), and mouse anti-MAP2 (1:500, Sigma). Secondary antibodies used were donkey anti-rabbit Alexa 488 (Invitrogen), donkey anti-mouse Alexa 546 (Invitrogen), and donkey anti-mouse Cy3 (Jackson ImmunoResearch Inc.). All secondary antibodies were applied at a dilution of 1:1,000.

### Tractography

Specimens for *ex vivo* MRI were fixed in paraformaldehyde and transferred to Fombin in a plastic syringe and mounted in a 16-mm coil (Rapid Biomedical, Würtsburg, Germany) in a 9.4-T MRI system (Agilent technologies, Yarnton, UK). Diffusion weighted data were recorded overnight using a spin echo sequence (TR = 4 s, TE = 18.17 ms, *b* = 1,250 s/mm^2^, 70 contiguous slices of 0.15 mm thickness, matrix = 192 × 192, FOV = 19.2 mm × 19.2 mm, ucl42 scheme, zero-filled to 256 × 256 before reconstruction). Tractography DSI studio DTI reconstruction was carried out using the following tracking parameters: termination index fa, Threshold, 90° Angular Threshold, step size 0.05, subvoxel seed position, trilinear direction interpolation, and the streamline (Euler) tracking Algorithm. The images were analyzed using DSI studio software (10). With this software, the diffusion information in each voxel is used for fiber tracing. Thus, the possibility for water molecules to move along axons and not across membranes during exposure to the magnetic field is to follow tracts from voxel to voxel. Several parameters have to be entered in the software before tracing. One critical parameter is the limit for angular reflection from the tract. In this study, this limit had to be gradually increased up to 90° to finally match the angle of the fibers in the dorsal root replant relative to the longitudinal axis of the spinal cord. Using automated software for tractography, it is important to change such parameters stepwise in order limit the risk for artifacts in the tracing.

The DSI software generates a 3D reconstruction from the stack of MRI images. In a virtual transverse section of the spinal cord, or dorsal root, a region of interest was used as the origin (seed) for the tracing of fiber tracts. The diffusion data from each voxel provide a description of the preferential movement of water molecules in the specimen. The diffusion is assumed to be restricted by axon membranes, but not restricted in the axoplasm along the fiber tracts. This generates a 3D image of the fiber tracts that stem from the so-called seed (the origin for the tracing). For the tracing of axons from the reimplanted dorsal root, we used a site within the root itself as seed (origin). We also performed tractography on the L3 region of the spinal cord where the dorsal root had been removed, but not reimplanted. As no root was available in the control specimen, the entire half of the spinal cord that was ipsilateral to the avulsed dorsal root was instead used as seed for the tractography.

## Results

**Figure 3:**
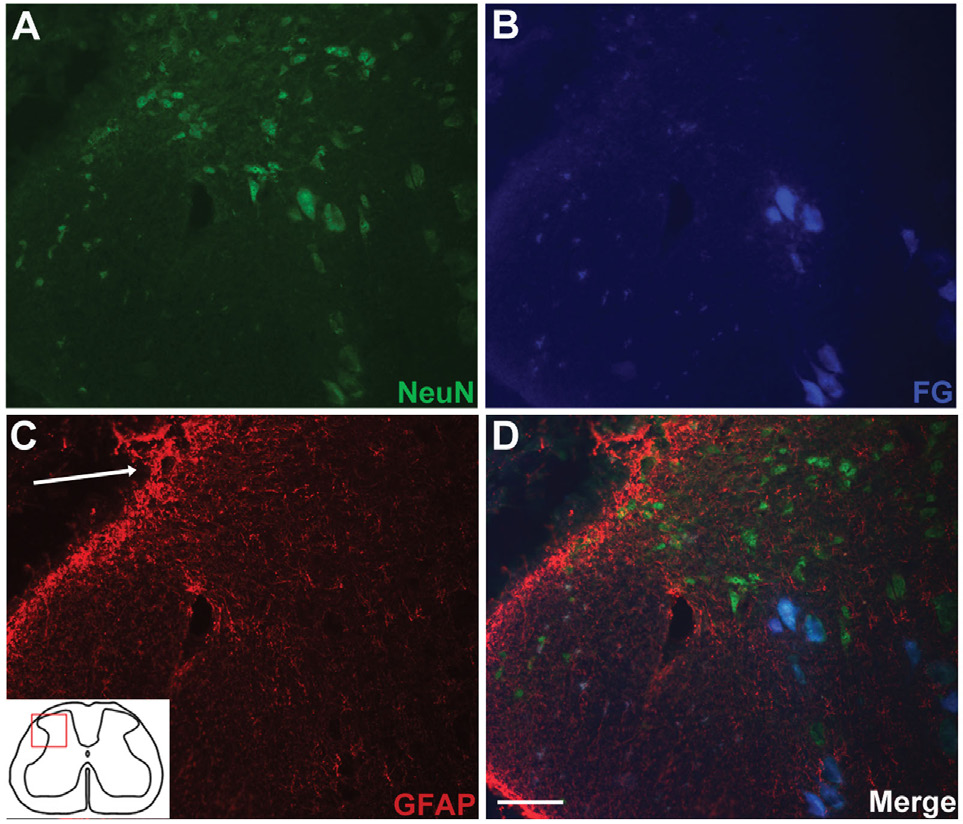
Retrograde tracing with FluoroGold (FG) indicates long distance extension of neuronal processes directly from spinal cord neurons to the sciatic nerve. Small populations of dorsal horn neurons [NeuN-positive; **(A)**] are colabeled with FG **(B,D)**. FG-positive neurons in the dorsal horn were typically found in the vicinity of the reimplantation site [**(C,D)**; arrow indicates implantation site]. Scale bar = 50 µm.

Anatomical assessment of reimplanted, ganglionectomised dorsal root tissue taken at 16 weeks post-injury revealed the presence of numerous small diameter axons when examining semi-thin plastic-embedded transverse sections taken adjacent to the ganglionectomy site (Figures 2C,D). Additionally, it was apparent that while almost all axons in the root were of a relatively small diameter (<3 μm), many of these were myelinated. While the small diameter of these axons suggested that they are likely to be *de novo* growth, they could possibly have been remaining fragments of degenerating axons following the avulsion and ganglionectomy. We therefore carried out retrograde tracing from the sciatic nerve using FG and examined transverse sections of the L5 spinal cord to verify the dorsal horn origin of some of these nerve fibers (Figure 3). Immunohistochemical analysis revealed that in each of the traced animals there were numerous examples of neuronal cell bodies (NeuN-positive) in the L5 dorsal horn which had incorporated the FG tracer (Figure 3), indicating that these cells must have a direct process which extended all the way to the sciatic nerve. Intriguingly, there were also many cases of FG-positive neurons being found in the lateral spinal nucleus (LSN). These FG-positive neurons occurred mainly in the vicinity of the tip of the reimplanted root in the spinal cord dorsal horn (Figures 3B,D).

**Figure 4:**
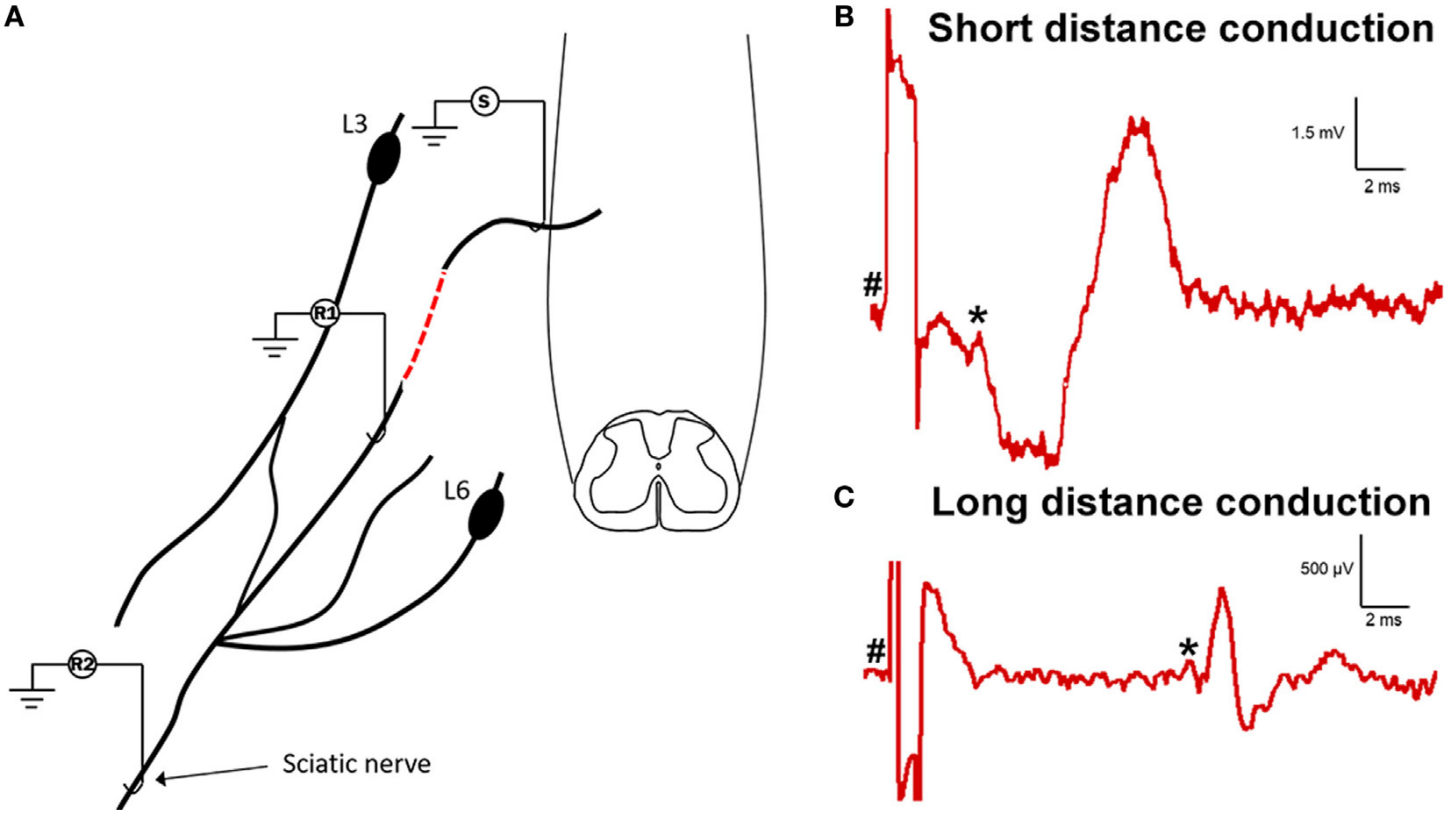
Electrophysiological assessment indicates functional viability of processes extending from spinal cord into dorsal root and distally to sciatic nerve. **(A)** Schematic drawing showing the experimental set-up for electrophysiological assessment of function in the ganglionectomised dorsal root/spinal nerve. (S), stimulation site; (R1), recording site on coapted spinal nerve (short distance conduction, 15 mm from S to R1); (R2), recording site on sciatic nerve (long distance conduction, 40 mm from S to R2). Dashed red line indicates the site of surgical repair, where L5 dorsal root transitions into the sensory part of L4 spinal nerve. **(B)** Example trace showing recording from proximal recording site (on spinal nerve). **(C)** Example trace showing recording from distal recording site (on sciatic nerve). Note the differing scales for each trace, indicating the increased amplitude and decreased latency for the short distance response. For panels **(B,C)**, ^#^ indicates onset of stimulus artifact and * indicates onset of evoked response.

In order to determine if the neural processes extending into and growing along the ganglionectomised dorsal root and spinal nerve were functional and could transmit electrical nerve impulses, we carried out an electrophysiological assessment of conduction 12–16 weeks post-injury (Figure 4A). To do this, we first stimulated the affected L5 dorsal root as it entered the spinal cord while recording any subsequently evoked activity at a site approximately 15 mm distal to this in the spinal nerve (Figure 4B). In six out of eight animals assessed, we saw varying degrees of evoked activity when recording at this site, indicating that not only had axons extended from the spinal cord out into this denervated tissue but that these neurites were functionally viable. In four of these animals (those not injected with FG), we then carried out further electrophysiological assessment in which we moved our stimulation site 10 mm distal to the previous site (to ensure there was no spread of stimulus to directly activate the spinal cord) and recorded any evoked activity in the sciatic nerve. In two of four animals assessed, we recorded evoked activity at this site (representative trace in Figure 4C), indicating that the newly formed neurites extending from the spinal cord were capable of conduction over long distances. One of the animals lacking long distance conduction had also displayed a lack of activity in the short distance experiment. The other animal exhibiting no short distance conduction was not assessed using this technique due to having received a sciatic nerve injection of FG. In both experiment types, the conduction velocity was indicative of conduction by thinly myelinated fibers (3.84 ± 0.3 m/s for short distance and 3.48 ± 0.4 m/s for long distance, *n* = 6 and *n* = 2, respectively). Interestingly, during electrophysiological experiments, we had noted that stimulation of the ganglionectomised dorsal root often resulted in a detectable muscle twitch response in the hind limb, even if stimulation was distal to the implanted dorsal root entry zone. This twitch response suggests that the fibers extending from the spinal cord into dorsal root and beyond could potentially be connected, either directly or indirectly, to motor neurons in the spinal cord (e.g., part of spinal reflex circuitry).

**Figure 5:**
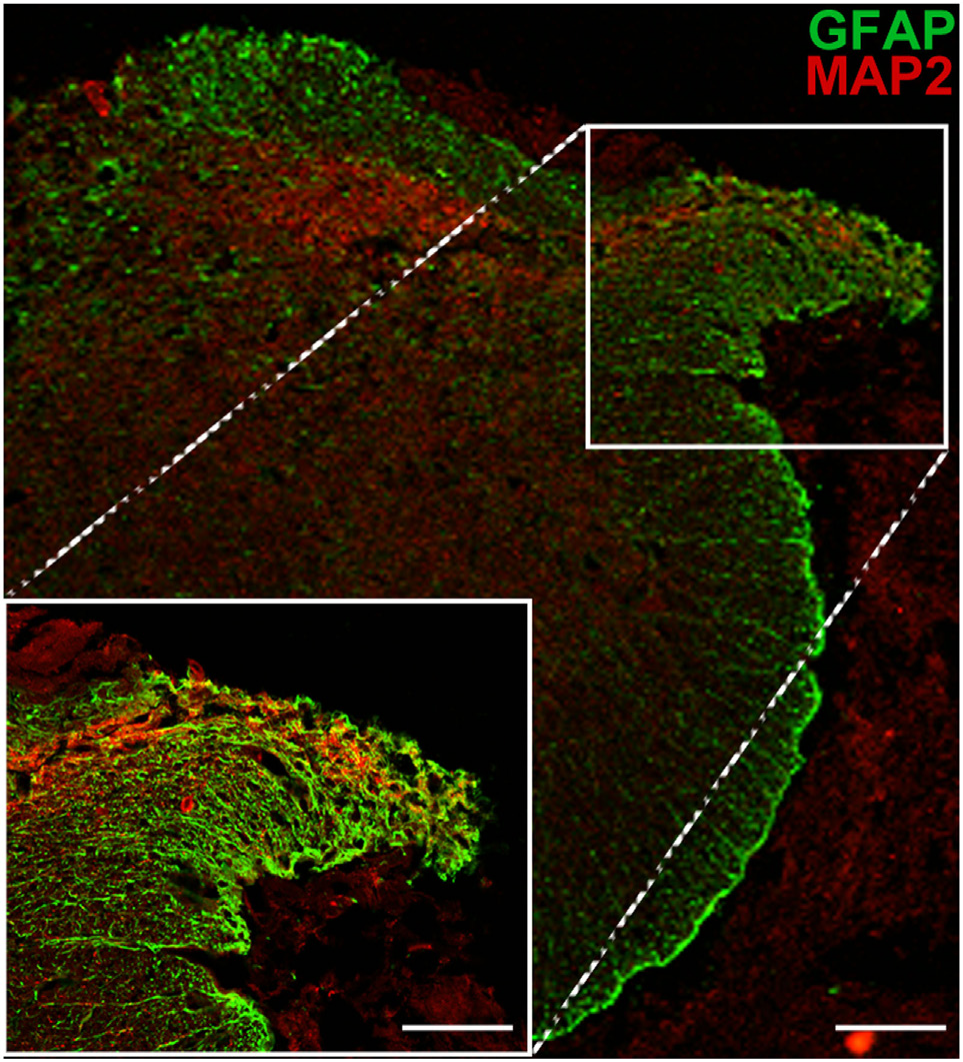
MAP2 immunopositivity indicates the dendritic origin of processes extending from the dorsal horn into the ganglionectomised dorsal root. Low magnification image shows the path of dendritic growth from dorsal horn into reimplanted dorsal root. High magnification inset highlights crossing of dendritic extensions from spinal cord to the reimplanted dorsal root. Scale bar = 70 µm for main panel and 50 µm for inset.

To further test the nature of the processes now identified as functional and extending from the dorsal horn into the reimplanted root and beyond, additional immunohistochemical analyses were carried out. We found that numerous longitudinal profiles crossing from the spinal cord into the implanted dorsal root could be identified as MAP2-positive (a dendritic marker) (Figure 5). The presence of a classical dendritic marker on these processes suggests that, following reimplantation of a ganglionectomised dorsal root, the dendrites of some neuronal cells in the adjacent dorsal horn are stimulated to extend toward and project along the denervated root, essentially becoming “dendraxons.”

**Figure 6:**
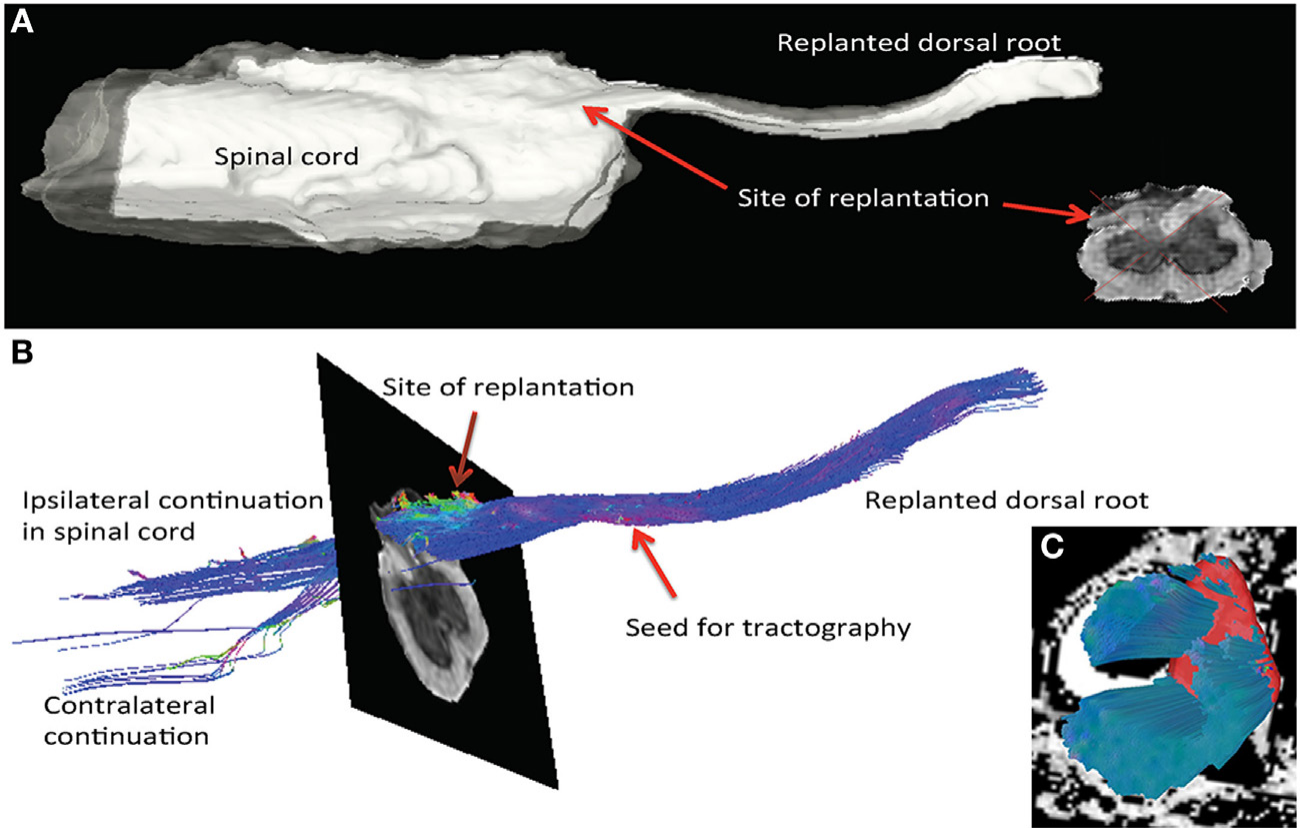
MRI tractography of spinal cord specimen from a rat subjected to dorsal root avulsion and ganglionectomy followed by reimplantation into the dorsolateral quadrant. **(A)** After perfusion fixation with paraformaldehyde, the specimen was analyzed by *ex vivo* imaging with a 9.4-T MRI scanner, allowing 3D reconstruction of the reimplantation site. Posttreatment of the images was performed in DSI studio software for tractography. **(B)** The seed for tractography was placed in the reimplanted root. The color code for fibers running longitudinal to the SC is blue, whereas fibers running perpendicular into the replant appear in green and a sagittal direction is represented in red. In this specimen, fibers could be followed from the SC into the dorsal root. Fibers entering the reimplanted root could be traced not only from the gray matter and ipsilateral dorsal column but also into the contralateral dorsolateral white matter. **(C)** At the site of the L3 rhizotomy, fibers are observed running longitudinally in the white matter, but none are exiting the cord (seed placed in ipsilateral white matter).

Further support of new growth from the spinal cord into the implanted dorsal root was obtained using a 9.4-T MRI scanner. There were anatomical signs of neurite extensions through the white matter and the root when a region of interest within the reimplanted root was used as the seed (origin) for tractography (Figures 6A,B). Fibers entering the root could be traced not only from the adjacent gray matter and ipsilateral white matter but also from the contralateral white matter to a lesser extent. Whether these fibers are continuous extensions directly into the root or are supernumerary axons from cells within the adjacent gray matter cannot be determined using this technique. In contrast to the findings at the reimplantation site, the L3 site (avulsion, but no reimplantation) showed no connection between the spinal cord and the avulsed dorsal root. Only, longitudinal fibers in the ventral, lateral, and dorsal funiculi were present (Figure 6C). The fiber content near the site of avulsion and the dorsal laminae of the dorsal horn seemed sparse. These results from the tractography, together with the histology and functional data, provide support of neurite extension and connectivity not only from the local spinal cord segment but also from long fiber tracts (Figure 6).

## Discussion

The present findings verify previous experimental (8) and human clinical surgical (1) results demonstrating that intrinsic spinal cord neurons can extend new (non-regenerative) processes into and beyond an implanted PNS conduit. Interestingly, although many of these spinal neurons were located in the dorsal horn, as previously described, retrogradely labeled neurons were also regularly observed in the white matter within the LSN. The role of the LSN is not entirely understood, but it has been implicated in both proprioception (11) and sensory processing, including nociceptive processing (12). It is difficult to predict the effect of activating such neurons due to the diverse range of nuclei to which they project (13), but their putative role in proprioception or sensory processing could provide a potential route for restoration of a spinal reflex arch. Importantly, not only can these intrinsic spinal cord neurons be transformed into projecting CNS/PNS neurons but such nerve cells are functional and can bypass or replace deleted or injured primary sensory neurons in the dorsal root ganglia. This depends on dendritic processes from spinal neurons extending into the periphery, not regenerating or regrowing previously injured axons.

A key addition to the previous and current anatomical findings was the use of electrophysiological experiments to demonstrate that the *de novo* extensions projecting from the spinal cord distally into the sensory spinal nerve and beyond were functionally viable. The activity recorded at both the sensory spinal nerve and the sciatic nerve was evoked antidromically, indicating a direct connection (i.e., no synapses involved) and allowing the mean conduction velocity of the potentials to be calculated. The rate of conduction was in accordance with that of small diameter, thinly myelinated axons. This adds to the previous findings where intrinsic neurons in the dorsal horn of the spinal cord were described to extend both myelinated and unmyelinated processes from CNS into PNS of the implanted nerve graft (8, 9). The electrophysiological demonstration of trans-synaptically evoked muscle contraction in the hindlimb while stimulating the *de novo* neurites in the reimplanted dorsal root suggests that there is an integration of these spinal cord neurons with segmental spinal cord circuits, and in particular with ventral horn motorneurons. This of course also supports the clinical finding of the returned muscle twitch reflex after a similar surgical procedure was performed in a human with root avulsion injuries (1). Whether the present findings represent newly established or spared contacts is not known. However, in previous studies, it was noted that some of the neurons that extended new processes to the implanted PNS conduit also retained normal or rostral projections (9). This suggests that the neurites grown into the periphery are aberrant processes.

A dorsal root injury is in effect a longitudinal spinal cord injury. The inability of DRG neurons to regrow after a dorsal root injury into the spinal cord is well documented (6). Although regrowth of injured dorsal root axons occurs in the root, it is impeded at the PNS–CNS interface (dorsal root entry zone) and regeneration is not possible. This is due to the inhibition of regeneration by CNS tissue but also to some degree the weak regenerating potential of the DRG neurons after a root injury (14). There are, however, situations where growth across the PNS–CNS interface is possible, such as from sensory neurons (DRG) in the immature animal (15). For example, regeneration occurred after root injury from the PNS into the spinal cord when the injury had been inflicted before there had been a development of a CNS–PNS transitional region at the root spinal cord junction. Obviously, there is an impediment to axonal growth once this CNS glia compartment in the root has been established. It was also noted that dendrites from dorsal horn neurons in those experiments had extended out from the spinal cord and into the dorsal root. In serial section analysis, it could be demonstrated that the same extension, which phenotypically resembled a dendrite in the CNS, appeared to have changed its phenotype and become a small myelinated axon in the dorsal root (15). These were very much similar to the myelinated nerve fibers seen in the implanted root in this experiment.

Previous root replantation experiments indicated that to re-establish function in humans after plexus root avulsion injury the strategy should be to “bypass” the CNS–PNS transitional region. This has been successful for motor recovery but not for sensory restoration. However, sensory recovery occurs when new neurite growth is provoked from spinal cord neurons into the periphery. Both strategies are similar, as neurons within the spinal cord grow from CNS to PNS. When inciting spinal cord neurons to extend new processes into the PNS, it is likely that not axons but dendrites would be recruited by the PNS conduit implanted into the dorsal horn. Dendrites have the ability to respond to extrinsic sensory stimuli, as well as to intrinsically produced molecules, and change their morphology. Secreted proteins such as neurotrophins and brain-derived neurotrophic factor (BDNF) can participate in shaping dendritic morphology (16). BDNF has a role in many aspects of neuronal development and plasticity (17) and can act as a positive regulator of dendritic growth. The upregulated production of BDNF (among other neurotrophic factors) by Schwann cells in the root following injury and axonal degeneration (18, 19), together with dynamic changes in the neuronal expression of neurotrophin receptors induced by spinal cord trauma (20, 21), could be of importance in the presently observed neurite extension from dorsal horn neurons. In a recent study, it was demonstrated that the axons of mature CNS neurons lack various molecules, such as integrins, which are essential for regeneration and possibly an important contributing factor to the weak regenerative response of CNS axons. In contrast, dendrites were shown to contain such pro-regenerative compounds (22).

Extension of supernumerary processes from spinal neurons into PNS conduits has previously been demonstrated only for motorneurons (23, 24). The present findings of MAP2 stained processes from the dorsal horn into the implanted root demonstrates that dendrites have extended into the implanted PNS conduit. It is now well established that spinal cord neurons can produce aberrant or supernumerary axons after injury. Such processes have been shown to extend from dendrites into the PNS and are termed dendraxons (23) or unusual distal processes (25, 26). It has also been shown that such neuronal processes can transmit impulses and contain transmitter substances for synaptic communication (24).

This basic science study has demonstrated the potential for new growth and plasticity, rather than regeneration, of sensory spinal cord neurons to reconnect to the periphery. Muscle twitch activity during stimulation of the reimplanted root revealed some form of direct or indirect connection between dorsal horn neurons extending processes into the reimplanted root and ventral horn motorneurons, thereby indicating the potential to re-establish a spinal reflex arch that could replace the function of lost primary sensory neurons. This example of plasticity and new dendritic growth, rather than axonal regeneration, could be considered as a possible underlying mechanism in studies and treatments for more “classical” transverse spinal cord injury, particularly in instances of providing some form of growth-permissive graft as part of a therapeutic intervention.

## Ethics Statement

This study was carried out in accordance with the United Kingdom Scientific Procedures Act (1986) and under the approved project license PPL 70/8032. Prior to Home Office approval the project license was also reviewed by an internal ethics panel at King’s College London.

## Author Contributions

TC, SM, MR, PD, and EB designed experiments. NJ, MA, PD, SM, MR, and TC performed experiments and data analysis. NJ, TC, PD, and MR wrote the manuscript. All authors reviewed and edited the manuscript.

## Conflict of Interest Statement

The authors declare they have no competing commercial or financial conflicts of interest.
